# Correlation of Strain Path, Texture, Twinning, and Mechanical Properties in Twinning-Induced Plasticity Steel during Wire Drawing

**DOI:** 10.3390/ma13102250

**Published:** 2020-05-13

**Authors:** Joong-Ki Hwang

**Affiliations:** School of Mechanical Engineering, Tongmyong University, Busan 48520, Korea; jkhwang@tu.ac.kr; Tel.: +82-51-629-1567

**Keywords:** twinning-induced plasticity steel, strain path, wire drawing, drawing direction, deformation twin, grain orientation

## Abstract

The effect of changing the strain path on texture development, twin kinetics, and mechanical properties in twinning-induced plasticity steel was investigated to understand twinning behavior in more detail. Among the various plastic deformation processes, the wire drawing process was selected to achieve the aims of the study. Specimens of cold-drawn TWIP steel wire under the same effective strain but with different crystallographic textures were successfully fabricated using the effect of the wire drawing direction. Electron backscatter diffraction results showed that the drawn wires using both unidirectional (UD) and reverse-directional (RD) wire drawing processes were characterized as duplex fiber textures of major <111> and minor <100>. It was found that the RD wire had a higher fraction of <111> component at both the center and surface areas compared to the UD wire, because the metal flow of the RD wire was beneficial for the development of a <111> orientation. The pronounced <111> crystallographic orientation of the RD wire activated the twinning rate and geometrically necessary dislocation density, leading to an increase in strength but a decrease in ductility. The strain path is as important as the amount of strain for strengthening the materials, especially those that are deformed by twinning.

## 1. Introduction

Twinning-induced plasticity (TWIP) steels have received significant attention as new advanced materials for possible application in the automotive industry due to their exceptional combination of strength and ductility [1,2]. Most researchers have attributed the high strength and ductility of TWIP steels to the deformation twins that are generated during the process of plastic forming because fine twins shorten the mean free path of mobile dislocations, known as the dynamic Hall-Petch strengthening effect [3,4,5,6]. Many studies concerning TWIP steels have therefore focused on the twinning behavior during plastic deformation in an attempt to understand the mechanism that leads to the exceptional strain hardening rate of TWIP steels [7]. In particular, the influence of grain orientation on the activity of deformation twinning during plastic deformation has received considerable attention, as twinning behavior depends strongly on grain orientation. The effect of grain orientation on deformation twinning behavior can be explained logically with Schmid factor analysis [8,9,10,11,12,13] via the use of uniaxial tensile and compressive tests. Gutierrez-Urrutia et al. [12] reported that the twinning activities complied with Schmid’s law under low strain in TWIP steel during a tensile test. It is well known that the main final texture under tensile stress is strong <111> and weak <100>, and grains that are close to the <111> orientation produce twins because the stress caused by twinning is lower than that of slip [13]. Yang et al. [9] suggested that grain rotation affects twinning behavior, based on results from tensile test experiments on Fe-33Mn-3Al-3Si TWIP steel using electron backscatter diffraction (EBSD) techniques and Schmid factor analysis. Their findings indicate that grain rotation leads to deformation twinning because twinning tends to occur in grains that are close to <111> orientation. In a similar manner, Meng et al. [10] suggested that grain rotation suppresses deformation twinning under compressive stress.

Twinning behavior is also dependent on the imposed stress states owing to the dependency of the twinning mechanism on the orientation of a grain [14,15,16,17]. Renard et al. [14] reported that twin density and morphology depend on the imposed stress state by comparing three different stress states, i.e., tension, rolling, and simple shear, with Fe-20Mn-1.2C TWIP steel. The results indicated that the presence of numerous, thin, and less parallel twins guarantees a higher strain hardening rate in TWIP steel, indicating that the stress state controls both the twinning behavior and mechanical properties. Nakada et al. [16] reported that deformation twinning behavior is significantly affected by the stress state in 316 stainless steels. For instance, one variant of twins forms mainly in cold-rolled specimens, while two variants tend to form in cold-drawn specimens. In addition, Hwang [17] reported that grain rotation to a <111> orientation encourages the formation of straight-type twins with larger lateral growth and more twin variants under the tensile stress. In contrast, grain rotation close to a <110> orientation under compressive stress suppresses the lateral growth of twins and the production of twin variants, and develops wavy-shaped twins because the <110> orientation is not a twinning-favorable region.

Based on the above literature and the experience of the author, strain path change can affect twin structures as well as dislocation structures [18,19]. Although the majority of studies have been conducted on twinning behavior under simple forming processes such as tension, compression, cold rolling, and wire drawing in TWIP steels, the relationship between strain path and twinning behavior has rarely been investigated. In particular, to understand the effect of strain path change on the mechanical properties and microstructure of a material, it is necessary to study the influence of different strain paths under the same effective strain. Several studies have reported that changes in the strain path can affect the evolution of the microstructure and mechanical properties of metals. Koohbor et al. reported the effect of deformation path changes on the static recrystallization behavior in a low carbon steel and pure aluminum during cold rolling and annealing processes [20,21]. They changed the rolling direction of materials and compared the kinetics of the recrystallization. They suggested that the changes in the rolling path significantly affect the static strain aging kinetics of low carbon steel and the recrystallization behavior of pure aluminum during the annealing process. Higginson and Sellars [22] also investigated the effect of strain path changes on the recrystallization kinetics of 316L stainless steel during the hot rolling process, finding that the change in rolling direction retards the recrystallization kinetics of stainless steel. 

To the best of the author’s knowledge, no studies have yet been dedicated to the investigation of the effect of strain path change on the microstructure and mechanical properties of TWIP steels under the same effective strain. Consequently, the effect of strain path changes on the twin kinetics and mechanical properties of TWIP steel is still poorly understood. Therefore, this study deals with the effect of strain path changes on the twin kinetics and mechanical properties in TWIP steel in order to understand twinning behavior in more detail. Of the various plastic deformation processes, the wire drawing process was selected to achieve the study objectives. Wire drawing is the most common currently used process for plastic forming in the wire and rod manufacturing industries [23,24]. In this study, wires were drawn using a two pass schedule at room temperature with a draw bench machine to produce unidirectional (UD) and reverse-directional (RD) wires. The drawing direction of the wire was changed every pass during the RD process. Carrying out this procedure meant that the two wires fabricated by the UD and RD process could have the same effective strain but different strain paths, textures, microstructures, and mechanical properties. A numerical analysis was also performed to evaluate the strain distribution of a drawn wire as associated with the drawing direction, and the mechanical properties of the drawn wire were correlated with the microstructures, especially twinning behavior, using EBSD techniques.

## 2. Experimental and Numerical Procedures

### 2.1. Experimental Procedure

#### 2.1.1. Casting and Hot Rolling of TWIP 

A steel with a designed chemical composition of Fe-17Mn-0.7C-1.5Cu (wt.%) was fabricated via vacuum induction melting. The chemical composition was analyzed using a spark optical emission spectrometer, and is listed in Table 1. The ingot was machined into a billet of 125 × 125 × 245 mm^3^ for hot rolling. After being soaking at 1200 °C for 12 h, the billet was hot-rolled directly into a plate with a final thickness of 20 mm at a final rolling temperature of 950 °C, and subsequently air cooled to room temperature to simulate the industrial production process of wires and rods. It was expected that the produced steel would be deformed by twinning and dislocation gliding, and martensitic transformation was suppressed during the room temperature plastic deformation owing to the value of calculated stacking fault energy (SFE), which was approximately 20.1 mJ/m^2^ [25,26] based on the thermodynamic models by Saeed-Akbari et al. [27] and Dumay et al. [28].

#### 2.1.2. Wire Drawing

The plate was fabricated into round bars (15 mm in diameter) using a lathe to perform the multipass wire drawing test. The round bars were drawn using the two different pass schedules: UD and RD wire drawing. In the case of the RD process, the drawing direction of the wire was changed at every pass, as shown in Table 2. Drawing tests were conducted at a constant drawing speed of 0.5 m/min with a semidie angle of 6°. A spray-type MoS_2_ lubricant was used prior to the wire drawing test. The specific die designs used in this test are presented in Table 2. The reduction of area (RA) per pass is calculated using the reduction ratio of the wire. The drawing strain (*ε*) of the wire is calculated using the change in the cross sectional area (*A*) or diameter (*d*) of the wire as follows:

(1) $$\varepsilon =\ln\frac{A_{0}}{A}=2\ln\frac{d_{0}}{d}$$

where *A*_0_ and *d*_0_ indicate the initial cross sectional area and diameter of the wire, respectively. 

#### 2.1.3. Microstructure and Mechanical Properties

The microstructures were characterized using EBSD and optical microscopy (OM) on the cross section of the specimen perpendicular to the wire drawing axis. EBSD observation was carried out using a field-emission scanning electron microscope equipped with a TSL system. Specimens for EBSD measurement were prepared by mechanical polishing using abrasive papers and diamond pastes, and next by polishing using colloidal silica slurry. The EBSD maps were acquired using a step size of 0.1 μm for the microstructural observation and 0.4 μm for the texture evaluation. The obtained EBSD data were evaluated using the TSL OIM commercial software version 8. The specimens were etched with 3% Nital for OM observation. 

The mechanical properties of the specimens were evaluated using a tensile test and the Vickers microhardness (HV) test. Due to the shape of the drawn wire specimens, round-type tensile specimens (5 mm in gauge diameter and 25 mm in gauge length) were used along the wire drawing and hot rolling axis. An Instron testing machine was used to pull the specimens at a strain rate of 10^−3^ s^−1^ with a mechanical extensometer at room temperature. To evaluate the ductility of the drawn wire, the RA value of wire was carefully measured after the tensile test [29]. Hardness was measured on the cross section of specimens perpendicular to the drawing axis with the test load varying from 0.5 to 1.0 kg over a constant holding period of 15 s

### 2.2. Numerical Modelling

To evaluate the strain distribution of the wire during the UD and RD processes, the finite element (FE) method with commercial metal-forming software DEFORM version 11.0 was used. A two-dimensional axi-symmetric module under isothermal condition was used, because the deformation of the wire occurs symmetrically and the drawing speed is low [30]. The required stress-strain curve for the input parameter in the FE modeling was acquired using a uniaxial tensile test [31]. To obtain more accurate numerical results, the effect of strain rate needed to be considered, because C-bearing TWIP steels are sensitive to the strain rate [32,33,34,35,36,37]. In other words, TWIP steels exhibited both negative and positive strain rate sensitivities with strain rate. Accordingly, the input stress-strain curve was modeled using the following for the current TWIP steel:

(2) $$\sigma =2174\varepsilon^{0.58}{\left(\frac{\overset{\cdot }{\varepsilon_{}}}{10^{-3}}\right)}^{-0.014}$$

The wire was assumed to be an isotropic material in this simulation, because hot-rolled steel was used as an initial specimen for the wire drawing test. A shear friction coefficient of 0.1 was selected for the wire-die interface; this was based on previous investigations [38,39,40]. To save on computational cost, half of the geometry was modeled because of the symmetrical constraints of the drawn wire. Finer meshes were applied to the surface area due to the higher strain in the surface area. Approximately 7000 rectangular-type meshes were used to obtain more accurate results. 

## 3. Results

### 3.1. Microstructural Evolution 

**Figure 1:**
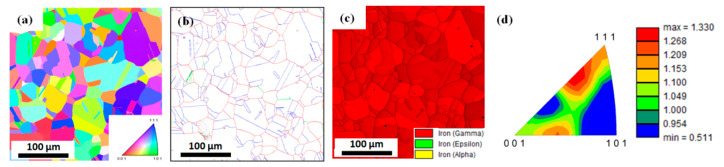
EBSD (**a**) IPF map, (**b**) grain boundary map, (**c**) phase map, and (**d**) texture of hot-rolled steel. Green, red, and blue lines represent low angle grain, high angle grain, and Σ3 twin boundaries, respectively, i.e., −15° > θ ≥ 2°, −θ ≥ 15°, −Σ3.

Figure 1 shows the microstructure and texture of the hot-rolled TWIP steel produced in this study. It is apparent that recrystallized grains (average size of 41.3 μm) were obtained after the hot rolling process. The phase map indicated a high stability in the austenite phase, as no other phases were observed (Figure 1c). A weak texture was also detected, as shown in Figure 1d. 

**Figure 2:**
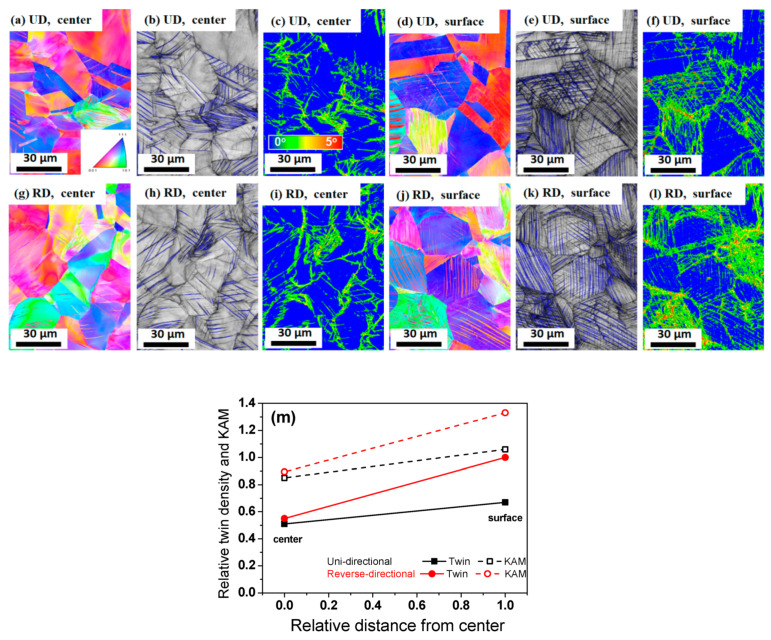
Comparison of IPF, IQ, and KAM maps at the strain of 0.26 between the two pass schedules: (**a**–**f**) UD wire, (**g**–**l**) RD wire, and (**m**) relative twin density and KAM value of the drawn wire.

Figure 2 compares the inverse pole figure (IPF) map, the image quality (IQ) map, and the Kernel average misorientation (KAM) map of the drawn wire at a strain of 0.26 subjected to UD and RD based on the EBSD techniques. To understand the twinning behavior of the two pass schedules in more detail, the Σ3 twin boundaries were compared using the EBSD. Boundaries with a misorientation angle of 57° <θ < 63° were considered as the Σ3 twin boundaries [41]. It should be noted that the Σ9 and Σ27 twin boundaries were ignored in this study because they rarely occur in TWIP steel during plastic deformation [42]. The blue lines were characterized as the Σ3 twin boundaries in the IQ maps [30]. Compared to the center area, a higher twin density was clearly observed at the surface area in both pass schedules. Interestingly, the RD wire had a slightly larger twin density compared to the UD wire, regardless of area. In order to obtain quantitative information on twinning behavior, the relative twin density was calculated and compared using the EBSD [43], as shown in Figure 2m. It should be noted that the twin density was compared using EBSD techniques, because all twins do not appear in the IQ map due to the limited resolution of the EBSD [30]. A difference in the twin density of the UD and RD wire was observed, indicating that a higher twin density was generated by the RD process throughout the entire cross section, compared to that of the UD process. The KAM value was also analyzed to confirm the different twinning behavior of the UD and RD wires, because misorientation mapping methods, such as calculation of the KAM value are useful for studying the features of plastic deformation [44]. It is well known that the KAM value reflects the geometrically necessary dislocation (GND) density [45,46]. A maximum angle of 5° was considered in calculating the KAM value. Obviously, the KAM of the surface area was much higher than that of the center area, regardless of the pass schedule. Additionally, a higher KAM was observed in the RD wire compared to the UD wire at the same area (Figure 2m), meaning that the wire that was deformed via the RD process had a larger GND density than the UD wire. 

**Figure 3:**
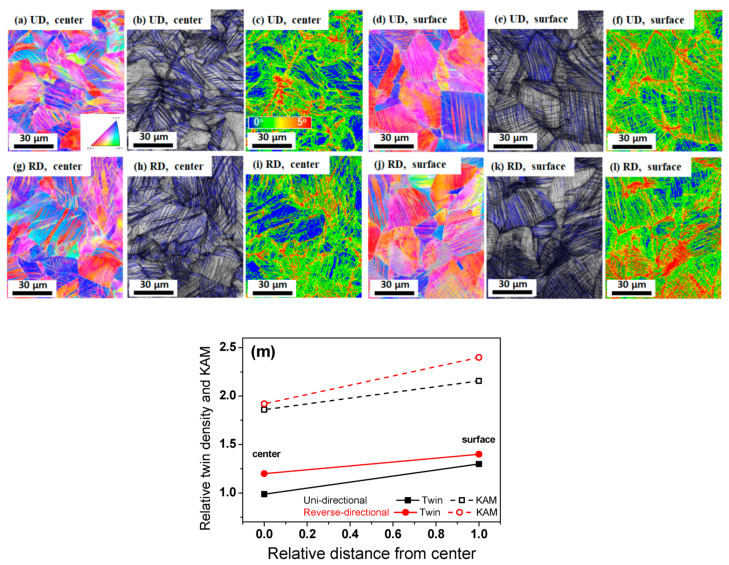
Comparison of IPF, IQ, and KAM maps at the strain of 0.5 between the two pass schedules: (**a**–**f**) UD wire, (**g**–**l**) RD wire, and (**m**) relative twin density and KAM value of the drawn wire.

Figure 3 shows the IPF map, IQ map, and KAM map of the drawn wire with an area at a drawing strain of 0.5 subjected to the UD and RD processes. The twin density and KAM values were much higher compared to the specimens produced at a drawing strain of 0.26 because of the greater accumulation of drawing strain. However, the trend of twinning behavior over a particular area and with a particular drawing direction was similar to that seen in the wire drawn at 0.26, as demonstrated quantitatively in Figure 3m. The RD wire had a higher density of deformation twinning and a higher KAM value, regardless of area; additionally, a higher fraction of twinning and KAM value were also observed at the surface area.

**Figure 4:**
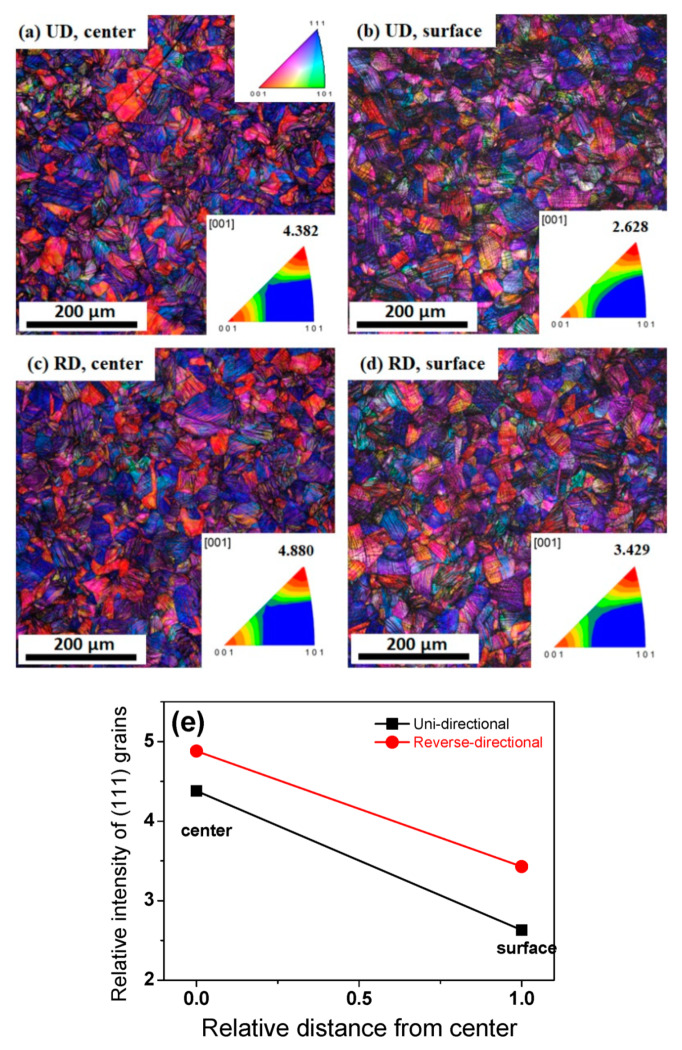
Comparison of (**a**–**d**) IPF maps and (**e**) relative intensity of <111> grains of drawn wire at a drawing strain of 0.5 with the area and drawing pass schedule.

To better understand the above results describing the characteristics of the microstructure, the texture evolution was examined. Figure 4 compares the texture of the two different pass schedules at a drawing strain of 0.5. It is well known that the IPF analysis is sufficient to evaluate the texture of drawn wire owing to the occurrence of fiber textures in the wire along the drawing direction during wire drawing [30,47]. A duplex fiber texture with a major <111> and minor <100> was observed, which is a well-known stable deformation texture for drawn metals with a FCC structure [47,48,49]. Figure 4e compares the relative intensity of the <111> grains of the drawn wires with the area and pass schedule. Clearly, the intensity of the <111> component was stronger at the center area than at the surface area, regardless of the drawing direction, due to the die geometry and the frictional stress occurring at the wire-die interface. This result is also commonly reported in FCC metals. The higher shear strain at the surface area of the drawn wire suppresses the ideal <111> texture component and promotes the formation of complex textures. Meanwhile, Figure 4e reveals the interesting finding that the RD wire had a stronger intensity of the <111> component at both areas in comparison with the UD wire. 

### 3.2. Mechanical Properties 

**Figure 5:**
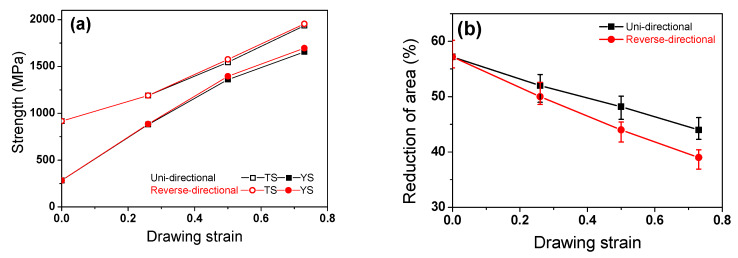
Comparison of (**a**) strength and (**b**) reduction in area with the pass schedules and drawing strain.

The tensile stress-strain curve of the hot-rolled TWIP steel displayed characteristics that are generally seen in C-added TWIP steels [31]. The strength of the RD wire was slightly higher than that of the UD wire, as shown in Figure 5a [50]. With regard to ductility, the elongation was similar between the two pass schedules with the same drawing strain; however, the RA of the RD wire was lower than that of the UD wire, as shown in Figure 5b. This result revealed that the RD wire had a lower ductility or formability compared to the UD wire, which is very different from the results obtained by Yoshida et al. [51,52]. Their findings showed that both Al wire and pearlitic steel wire fabricated by the RD process exhibited a higher ductility than those produced via the UD process.

**Figure 6:**
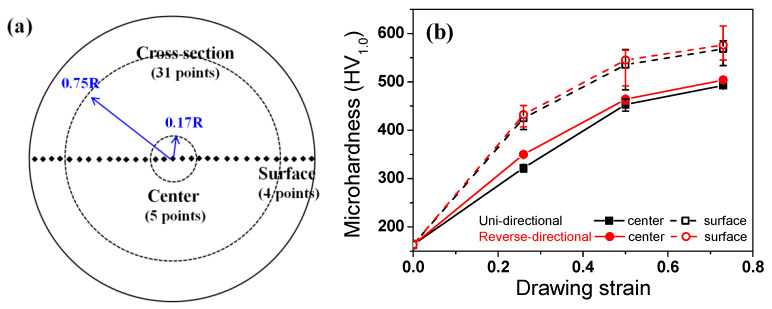
(**a**) Schematic description showing the positions at which hardness was measured on a cross-section of the wire, and (**b**) Comparison of the variation in the measured hardness of the two pass schedules with drawing strain.

To determine the mechanical properties of the UD and RD wire with area, the hardness was also measured. Figure 6a schematically represents the measurement positions of center and surface areas. The hardness increased with drawing strain, and the surface area had higher hardness values compared to the center area, regardless of the pass schedule, as shown in Figure 6b. It is interesting to note that the RD wire had slightly higher hardness in comparison to the UD wire at both the center and surface areas, although the difference was small. 

### 3.3. Analysis of Strain and Stress 

**Figure 7:**
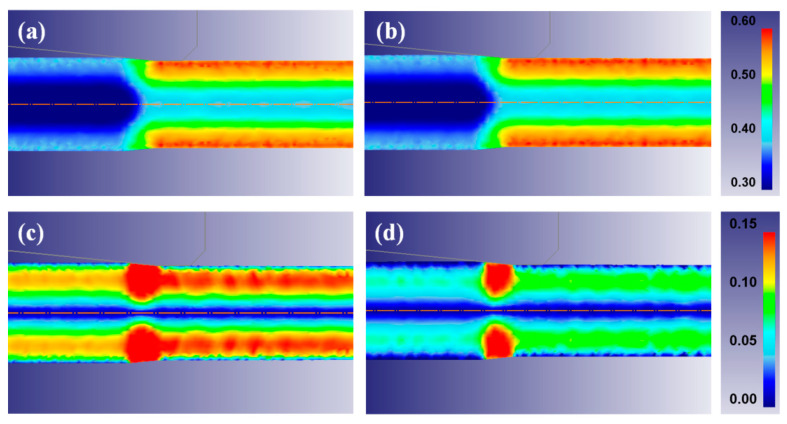
Comparison of the effective strain contour of (**a**) UD and (**b**) RD, and the shear strain contour of (**c**) UD and (**d**) RD at a drawing strain of 0.39.

**Figure 8:**
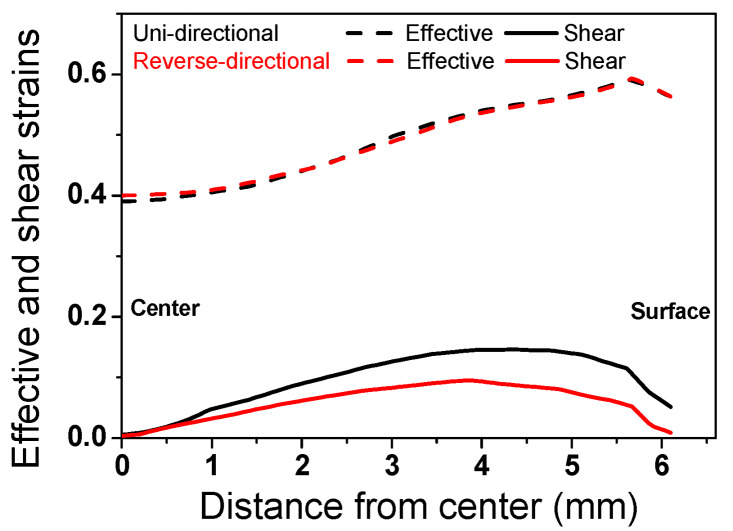
Comparison of the profiles of effective and shear strains along the radial direction of the wire at a drawing strain of 0.39.

**Figure 9:**
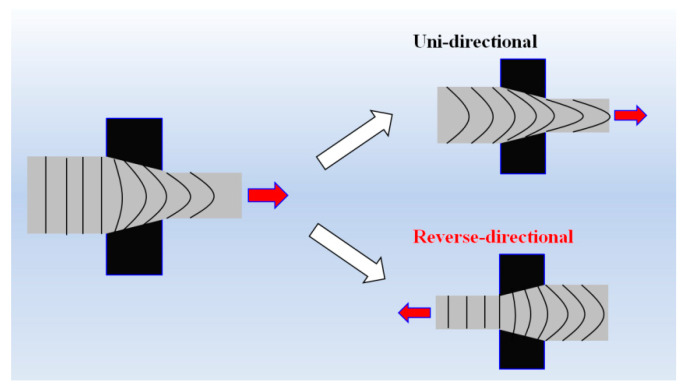
Schematic comparison of the metal flow between the UD and RD process.

To better understand the unexpected development in texture between the UD and RD wire, an FE analysis was conducted. Figure 7 shows the contour maps of the effective and shear strains at a drawing strain of 0.39. A similar effective strain was observed in both the UD and RD wire along the radial direction. In contrast, the shear strain along the radial direction of the wire was very different with the two pass schedules, as shown in Figure 8. The shear strain of the RD wire was lower than that of the UD wire, since a shear strain was suppressed when the drawing direction was changed [50,51]. This result may explain the higher fraction of <111> orientation at the surface area of the RD wire, because it is well known that shear strain suppresses the <111> texture component during wire drawing. However, it cannot explain the higher fraction of <111> components at the center area of the RD wire. Based on the change in the shape of mesh, metal flows were schematically drawn, as shown in Figure 9. It is well known that metal flow reflects the history of the strain path or stress state [53]; this can make it easier to understand the deformation behavior of multipass forming processes. The metal flow of the RD wire was more uniform in comparison with the UD wire, leading to a higher intensity of <111> texture components in the RD wire. A reduction in the shear strain of the RD wire was clearly exhibited in the surface area. In other words, different metal flows occurred depending on the strain path despite being subjected to the same effective strain, resulting in different textures. Note that the texture gradient of the RD wire decreased along the radial direction of the wire, as shown in Figure 4, because of the uniform metal flow.

## 4. Discussion

One interesting result of this study is the slightly higher hardness (Figure 6) and lower ductility (Figure 5) of the RD wire in comparison with the UD wire, which occurred despite the similar effective strain of the two wires (Figure 7). This result is not easy to understand at first. For instance, Yoshida et al. reported a comparison of the mechanical properties of UD and RD wire using pearlitic steel [51] and Al wire [52]. They showed that the RD wires exhibit a lower strength and a higher ductility compared to the UD wires. This inconsistency of TWIP steel and other metals could be related to their microstructure development and the strengthening mechanisms during wire drawing. Accordingly, the unexpected results from TWIP steel can be explained through an evaluation of the microstructure and the strengthening mechanism of TWIP steel. The RD wire had higher intensity of a <111> component in comparison with the UD wire, regardless of position. The higher <111> orientation activated deformation twinning during the plastic deformation. Hwang et al. [30] demonstrated an orientation dependency in twinning behavior during the wire drawing process using the Schmid factor analysis. They derived favorable and unfavorable grain orientations for twinning and slip under the assumption of equal critical resolved shear stresses for slip and twinning [12]. According to their findings, deformation twinning easily occurred close to the grains of the <111> and <110> orientations under tensile stress. 

**Figure 10:**
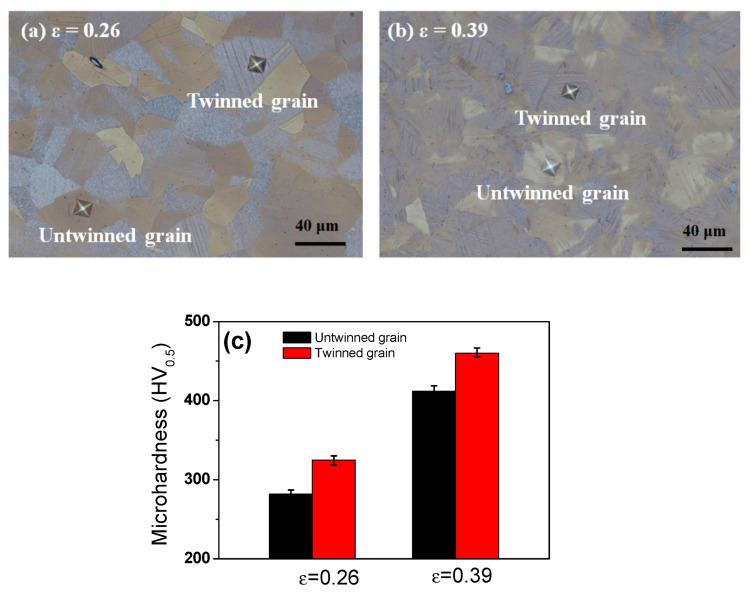
Image of dents in twinned grain and untwined grain during the microhardness test at drawing strains of (**a**) 0.26 and (**b**) 0.39, and (**c**) a comparison of the microhardness values of the twinned grain and the untwinned grain.

Figure 10 compares the hardness value of the twinned grain and the untwinned grain at drawing strains of 0.26 and 0.39. Regardless of the drawing strain, twinned grains had higher hardness values compared to untwinned grains, since the occurrence of deformation twinning during plastic deformation can divide the austenite grains, restricting the dislocation movement and increasing the critical resolved shear stress via the grain refinement effect, resulting in strengthening. Moreover, it was also reported that the <111> component increases the strength and decreases the ductility in FCC materials compared to <100> and <110> orientations under tensile stress [52,54,55]. 

In summary, the change in wire drawing direction produced more uniform metal flow along the radial direction of the wire in comparison with the unidirectional wire drawing. The uniform metal flow over the entire area encouraged the development of the <111> orientation in the drawn wire, regardless of the area during the wire drawing. Schuman et al. [56] also reported that the RD wire had a higher fraction of stable drawn texture compared to the UD wire in low carbon steel. The stronger <111> density in the RD wire encouraged deformation twinning during wire drawing, because the main stress state during wire drawing is tension, which leads to an increase in the strength of the RD wire. In case of the surface area, the twinning behavior of the RD wire was more prevalent by the two mechanisms. First, the higher amount of a <111> texture component encouraged deformation twinning because the main stress state is also tension at the surface area. Second, the twinning behavior is highly related to the strain path effect. Although the RD wire had a lower final shear strain at the surface area compared to the UD wire (Figure 6), the strain path of the RD wire was more complex due to the directional change of the specimen during wire drawing. Hwang et al. [30,50] reported that a complex strain history can activate deformation twinning, regardless of the grain orientation during the plastic forming process, leading to the higher twin density of the RD wire.

Meanwhile, Yoshida et al. [51] reported that the TS of pearlitic steel wire fabricated by the RD process is lower than that fabricated by the UD, which is very different from the results with TWIP steel presented in this study. To the best of the author’s knowledge, dislocation hardening (*σ_d_*) and grain refinement hardening (*σ_g_*) are the main strengthening mechanisms in both steels as follows:

*σ_t_* = *σ*_0_ + *σ_s_* + *σ_p_* + *σ_d_* + *σ_g_* (3)

where *σ_t_*, *σ*_0_, *σ_s_*, and *σ_p_* refer to the total stress, the lattice friction stress, the solid solution hardening effect, and the precipitation hardening effect, respectively. *σ*_0_, *σ_s_*, and *σ_p_* were not directly related to the different hardening behavior in between UD and RD wires. In the pearlitic steel, dislocation density decreased when the drawing direction was changed, owing to the rearrangement of dislocation stemming from the strain path change or lower shearing stress that occurred during the RD process. It is well known that dislocation density is highly dependent on the shear strain during the plastic deformation process [57]. As shown in Figure 8, the RD wire had a lower shear strain compared to the UD wire. The grain refinement effect was almost same between the RD and UD wire, since the interlaminar spacing of the pearlitic steel was similar in both wires. Therefore, the strength of the RD wire was lower than that of the UD wire in the pearlitic steel. For TWIP steel, the grain refinement effect caused by deformation twinning increased when the drawing direction was changed. The higher twin density and GND density of the RD wire (Figure 2 and Figure 3) clearly supports the suggested strengthening mechanism. Consequently, the increase in the strength of the RD wire compared to the UD wire was mainly associated with the grain refinement effect that was caused by deformation twinning. The author believes that dislocation density of the RD wire also increased because the formation of deformation twin increased the activation of dislocations. In this respect, additional research is necessary. In this study, the higher twin and GND density of the RD wire was systematically explained using a comparison of metal flow and texture development between the UD and RD drawing process. It is worth noting that the effect of change in the strain path on the mechanical properties of a material should be taken into account in the design of industrial forming processes. 

## 5. Conclusions

Specimens of cold-drawn TWIP steel wire with the same effective strain but different crystallographic orientation were successfully fabricated using the directional effect during wire drawing. The strain path, texture, deformation twinning, and mechanical properties were evaluated and correlated, leading to the conclusions given below:

1. The results of the EBSD analysis highlighted that the drawn UD and RD wires were characterized as a duplex fiber texture of major <111> and minor <100>. However, the texture development differed slightly according to the pass schedule; the RD wire exhibited a higher intensity of <111> components in both the center and surface areas compared to the UD wire, because the metal flow of the RD wire was beneficial for the development of a <111> orientation. 

2. The pronounced <111> crystallographic orientation of the RD wire enhanced the twinning rate and GND density, leading to an increase in strength but a decrease in ductility. The increase in the strength of the RD wire compared to the UD wire was mainly associated with the grain refinement effect brought about by deformation twinning.

3. The strain path is as important as the amount of strain for strengthening the metals, especially when deformed by twinning, because the strain path controls the metal flow and the development of texture in materials during the plastic forming process.

## Figures and Tables

**Table 1:** Detailed chemical composition (wt.%) and calculated SFE of the steel examined in this study.

C	Mn	Cu	Fe	SFE (mJ/m^2^)
0.72	16.94	1.52	Bal.	20.1

**Table 2:** Process conditions for the wire drawing test used in this experiment.

Diameter of Drawn Wire (mm)	No. of Passes	RA per Pass (%)	Drawing Direction		Total RA (%)	Drawing Strain
			Unidirectional (UD)	Reverse-Directional (RD)		
15.0	-	-	-	-	0	0
14.0	1	12.8	Forward	Forward	12.8	0.13
13.18	2	11.3	Forward	Reverse	22.7	0.26
12.30	3	12.9	Forward	Forward	32.7	0.39
11.63	4	10.5	Forward	Reverse	39.8	0.50
10.97	5	11.1	Forward	Forward	46.7	0.62
10.40	6	10.1	Forward	Reverse	51.9	0.73
